# Identification of hub genes and therapeutic drugs in osteonecrosis of the femoral head through integrated bioinformatics analysis and literature mining

**DOI:** 10.1038/s41598-023-39258-4

**Published:** 2023-07-24

**Authors:** Lan Tang, Bin Li, Qiuming Su, Xi Chen, Rongxin He

**Affiliations:** 1 Department of Orthopedic, The Second Affiliated Hospital, Zhejiang University School of Medicine, #88 Jiefang Road, Hangzhou City, 310001 Zhejiang Province People’s Republic of China; 2 Key Laboratory of Motor System Disease Research and Precision Therapy of Zhejiang Province, Hangzhou City, Zhejiang Province China; 3 Department of Hepatopancreatobiliary Surgery, The First People’s Hospital of Kunming, Calmette Hospital, Kunming City, Yunnan Province China; 4 Department of Epidemiology and Statistics, School of Public Health, Medical College, Zhejiang University, Hangzhou City, Zhejiang Province China

**Keywords:** Computational biology and bioinformatics, Biomarkers, Diseases

## Abstract

Osteonecrosis of the femoral head (ONFH) is a multifactorial disease leading to severely limited function. By far, the etiology and pathogenesis of ONFH are not fully understood, and surgery is the only effective way to treat ONFH. This study aims to identify hub genes and therapeutic drugs in ONFH. Two gene expression profiles were downloaded from the gene expression omnibus database, and the hub genes and candidate drugs for ONFH were identified through integrated bioinformatics analysis and cross-validated by literature mining. A total of 159 DEGs were identified. PTGS2, LRRK2, ANXA5, IGF1R, MCL1, TIMP2, LYN, CD68, CBL, and RUNX2 were validated as 10 hub genes, which has considerable implications for future genetic research and related research fields of ONFH. Our findings indicate that 85 drugs interact with ONFH, with most drugs exhibiting a positive impact on ONFH by promoting osteogenesis and angiogenesis or inhibiting microcirculation embolism, rather than being anti-inflammatory. Our study provides novel insights into the pathogenesis, prevention, and treatment of ONFH.

## Introduction

Osteonecrosis of the femoral head (ONFH) is a multifactorial disease with an unknown etiology, characterized by loss of integrity of the subchondral bone^1^. It mainly affects men aged 30–50 years and involves more and more youngers^1,2^. Despite the identification of corticosteroid use, alcohol, sickle-cell disease, and trauma as the major risk factors for ONFH, its pathogenesis remains incompletely understood^2,3^. Surgery is the only effective way to treat ONFH at present^4^. In the USA, approximately 10% of hip replacements are due to ONFH, and in the UK, ONFH is the third most common indication for total hip replacements in people under 50, which causes a heavy socioeconomic burden^1,4^. Effective drug therapies have not been established^5^. Therefore, identifying novel biomarkers and therapeutic drugs involved in the ONFH is of great significance in exploring efficient treatment strategies.

Over the past two decades, few researchers have focused on exploring the mechanism of ONFH at the gene level^6^. Abnormalities in some genes, such as plasminogen-activating inhibitor-1 and nitric oxide synthase 3, have been proven to weaken the osteogenic differentiation capacity of bone marrow mesenchymal stem cells or disrupt angiogenesis, leading to the collapse of the femoral head surface^7,8^. However, the specific molecular mechanism of ONFH has not yet been clarified, and drug discovery has had little success. Recently, gene chips and high-throughput sequencing technologies have been widely used in the screening of disease-causing genes and have achieved practical and reliable results^9^. A new drug prediction method, drug-gene-disease triangulation based on literature mining, has been confirmed to be robust and reliable for identifying new drug candidates^10^.

In this study, we analyzed two gene expression profiles (Table 1) extracted from the National Center for Biotechnology Information Gene Expression Omnibus (GEO) database, which is a free global database of sequencing results. DEGs were identified by using the online tool GEO2R. Subsequently, the function of DEGs was determined by the Gene Ontology (GO) and Kyoto Encyclopedia of Genes and Genomes (KEGG) pathway enrichment analysis. With the help of Cytoscape, ten hub genes were selected and then validated by ROC analysis. Finally, the targeted drugs for ONFH were identified through the Drug-Gene Interaction Database (DGIdb) and cross-validated by integrated literature mining. Our study figured out the drug-gene-disease correlation to generate new insights into the prevention and treatment of ONFH.

**Table 1:** Detailed information on the GEO microarray profiles.

Profile	Organism	Sample source	Technology type	Cases	Controls	Platform
GSE123568	Homo sapiens	Peripheral serum	In situ oligonucleotide	30	10	GPL15207
GSE74089	Homo sapiens	Hip cartilage	In situ oligonucleotide	4	4	GPL13497

## Results

### Identification of DEGs in ONFH

**Figure 1:**
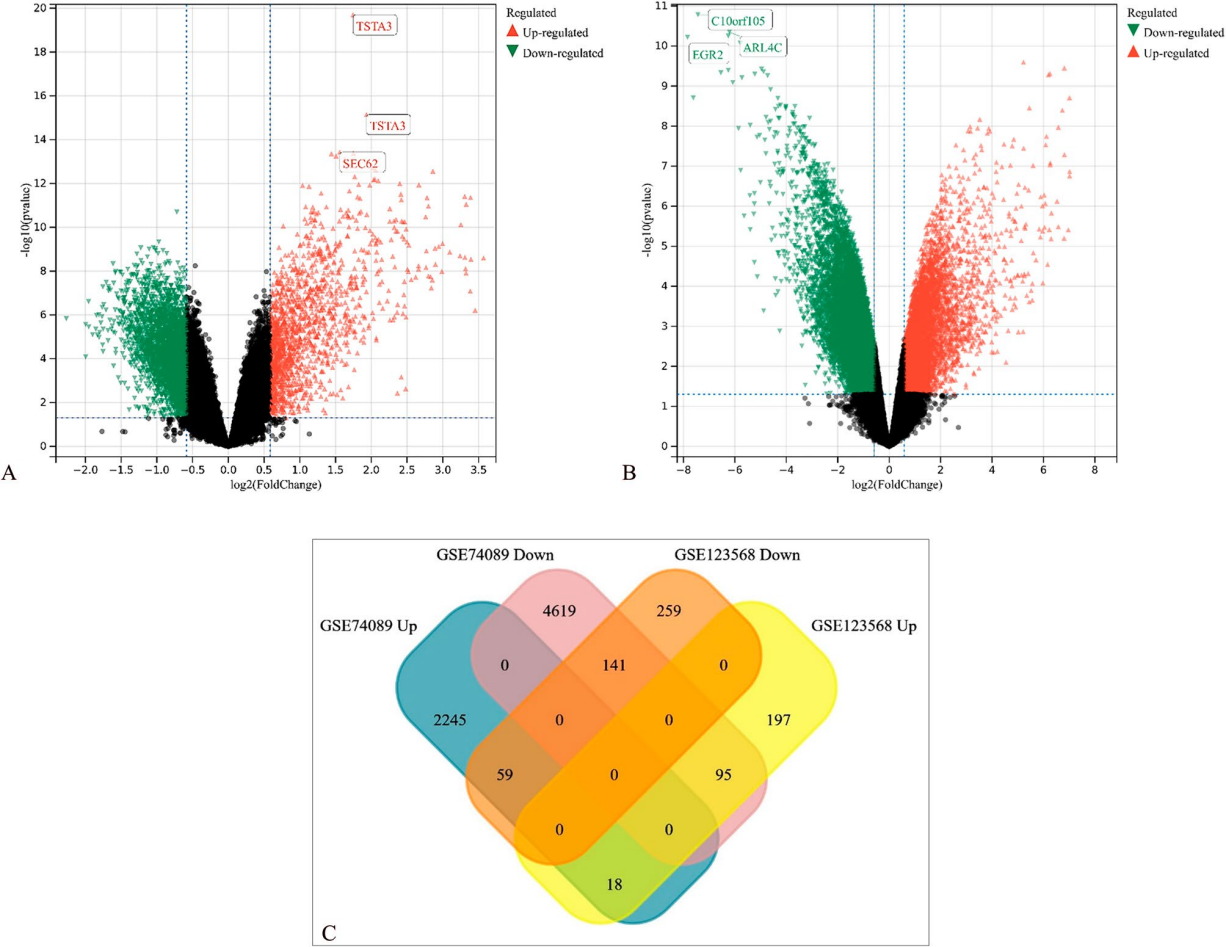
Differentially expressed genes (DEGs) in ONFH patients. The volcano plots were constructed based on (**A**) GSE123568, and (**B**) GSE74089. The green points indicated the down-regulated DEGs, the red points indicated the up-regulated DEGs, and the black points indicated genes with no significant differences under the cutoff criteria: adjusted *P*-value < 0.05 and | log2^FC^ |> 1. (**C**) Venn diagram showed the overlap of DEGs in 2 datasets.

Following dataset analysis, 769 DEGs were identified in the GSE123568 dataset, including 310 up-regulated and 459 down-regulated genes (Fig. 1A). A total of 7177 DEGs, including 2322 up-regulated and 4855 down-regulated genes, were identified in the GSE74089 datasets (Fig. 1B). 95 genes were verified to be highly expressed in blood and lowly expressed in cartilage, while 59 genes showed the opposite pattern (Fig. 1C). We finally found 18 up-regulated and 141 down-regulated genes in both datasets, as shown in the Table 2 and the Venn diagram (Fig. 1C). The expression levels of these DEGs were visualized in the form of heatmaps, as provided in Supplementary Fig. S1.

**Table 2:** Information on the identified DEGs.

DEGs	Gene names
Up-regulated	MYH10, RAP1GAP, ARG1, SLC4A1, FHL2, ENSA, GSPT1, ST6GALNAC4, SLC14A1, TFR2, WNK1, C1orf116, ANK1, RAB2B, TK1, CITED2, CEACAM6, SPTB
Down-regulated	TGFBI, PELI1, ARRDC3, PTGS2, VCAN, SGK1, RNF144B, IL17RA, S1PR3, TSHZ3, NID1, LOC100131541, SKAP2, RGS2, TET2, MPZL3, MXD1, VMP1, ABHD2, EFHD2, TREM1, KCTD12, DOCK8, SLC31A2, JMJD1C, FAM49A, OGFRL1, THBD, PID1, NAGA, FAM198B, MAFB, PLEKHO1, IKBIP, SPAG9, RIN2, PKN2, LRRFIP1, ZFP36L2, GPR155, FUCA2, CHST15, WLS, OSBPL8, ADRBK2, FRY, ASAP1, DENND3, EGLN1, ANP32A, RAB31, ATP11A, ANPEP, ABHD5, WIPF1, LYST, FAM49B, SPOPL, LAMP2, BACH1, FAM46A, SRPK2, CBL, RELL1, TIMP2, EPB41L3, FCHO2, NAMPT, CFLAR, CD68, ITPRIPL2, RHOQ, SVIL, SMAP2, NBPF1, ARHGAP27, KLF4, RHOB, MCL1, PPFIA1, CPPED1, PIGX, CD46, RNF130, SLC6A6, FAM174A, TXNDC5, NHS, MTMR11, CD14, ARHGAP9, FNDC3B, GLIPR1, KCNJ15, RUNX2, PTPRE, MPPE1, MSL1, QKI, BID, LRRK2, WDFY3, CAMK2G, MOSPD2, TMCC1, DMXL2, GNG11, ABHD3, TMEM154, IL13RA1, RICTOR, PPT1, CTBS, ATP6V1B2, SLC15A4, ZNF117, HIP1, IMPDH1, LYN, TSEN34, NOD2, SULF2, IGF1R, RPS6KA5, PDK4, BASP1, ATP6V1A, CXCL5, DUSP1, PSAP, RNF111, FAM129A, MSN, CEP63, PDE4B, IFNGR2, KCNE3, SMPDL3A, ANXA5, TACC1, PFKFB4

*DEGs* differentially expressed genes.

### Functional enrichment analysis of DEGs

**Figure 2:**
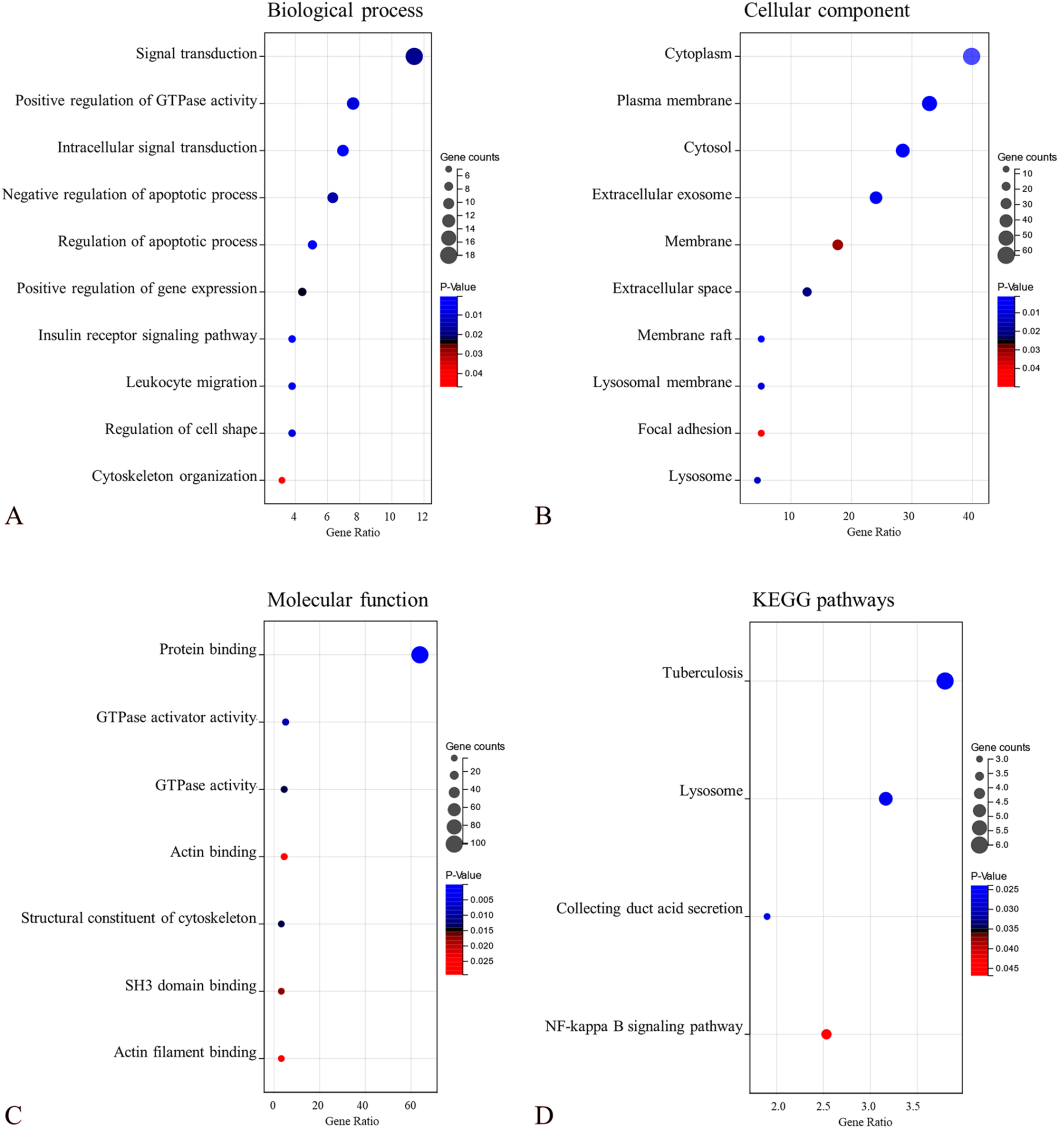
Bubble map for GO and KEGG pathway analyses of DEGs. The top 10 or all (if less than 10) significant items of the GO and KEGG pathway enrichment analysis were illustrated in (**A**) biological process, (**B**) cellular components, (**C**) molecular function, and (**D**) KEGG pathways. *P* < 0.05 was considered statistically significant.

To investigate the biological functions enrichment of DEGs, the 159 DEGs obtained above were then analyzed by DAVID. GO BP analysis showed that these 159 DEGs were markedly enriched in signal transduction, positive regulation of GTPase activity, intracellular signal transduction, and negative regulation of apoptotic process (Fig. 2A). GO CC analysis revealed that the DEGs were mainly involved in cytoplasm, plasma membrane, cytosol, and extracellular exosome (Fig. 2B). For MF analysis, DEGs were mainly enriched in protein binding, GTPase activator activity, GTPase activity, and actin binding (Fig. 2C). Regarding KEGG pathways, DEGs were significantly enriched in tuberculosis, lysosome, collecting duct acid secretion, and NF-κB signaling pathway (Fig. 2D). More details were provided in Supplementary Table S1.

### Hub gene determination and validation

**Figure 3:**
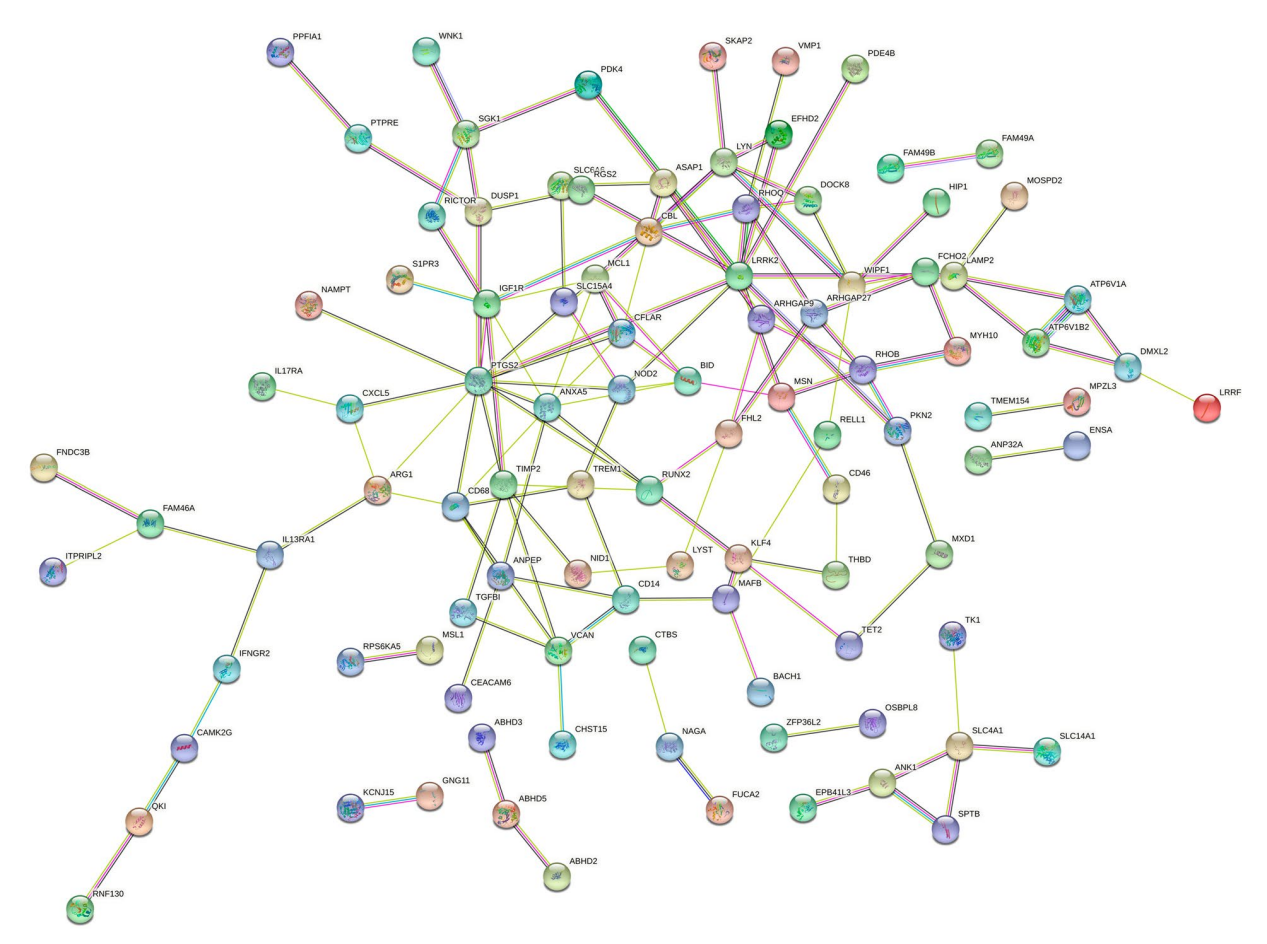
PPI network of 159 DEGs. Disconnected nodes had been hidden in the network.

The PPI network of the DEGs in ONFH was constructed in the STRING database. 158 nodes and 137 edges were established, and the PPI enrichment *P*-value was 6.9e-8 (Fig. 3). After then, a total of four clusters of functional modules were identified using Cytoscape software by the MCODE plugin, as shown in Supplementary Fig. S2.

**Figure 4:**
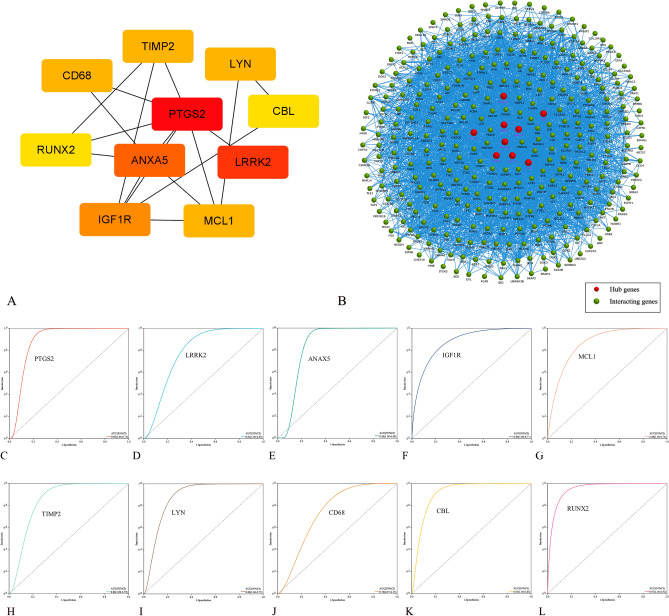
PPI network and ROC curves of the hub genes. (**A**) The PPI network of the hub genes with each other. The closer to red, the higher the Degree score. (**B**) The PPI network of the hub genes and interacting genes. Disconnected nodes had been hidden in the network. The ROC curves of (**C**) PTGS2, (**D**) LRRK2, (**E**) ANXA5, (**F**) IGF1R, (**G**) MCL1, (**H**) TIMP2, (**I**) LYN, (**J**) CD68, (**K**) CBL, and (**L**) RUNX2.

Ten genes (PTGS2, LRRK2, ANXA5, IGF1R, MCL1, TIMP2, LYN, CD68, CBL, and RUNX2) with the highest scores in the Degree, Bottleneck, and MCC algorithms were identified as the hub genes (Table 3). All the hub genes were down-regulated DEGs (Table 2). The PPI network showed 17 interactions of the hub genes with each other, the average local clustering coefficient was 0.507, and the PPI enrichment *P*-value was 6.4e-07 (Fig. 4A). In addition, an interaction network of the hub genes and their related genes was conducted in FunRich software. A total of 352 nodes and 7316 edges were established, and the PPI enrichment *P*-value was lower than 1.0e-16 (Fig. 4B, Supplementary Table S2). The KEGG pathway analysis showed that EGFR tyrosine kinase inhibitor resistance, apoptosis (multiple species), and chronic myeloid leukemia have the top 3 large enrichment effects in the network (Supplementary Table S3).

**Table 3:** The topology analysis results of 10 hub genes.

Gene	Degree	MCC	BottleNeck	Betweenness	Stress	Closeness
PTGS2	26	28	54	2355	277,936	36.7
LRRK2	20	10	35	1902	205,280	34.3
ANXA5	16	24	5	293	22,080	31.0
IGF1R	14	11	5	424	31,320	30.1
MCL1	12	19	5	337	44,928	29.3
TIMP2	12	7	3	431	34,736	29.1
LYN	12	6	6	463	55,984	26.9
CD68	12	8	6	341	70,928	28.7
CBL	10	5	3	270	19,952	27.6
RUNX2	10	6	7	624	69,792	29.6

In the ROC analysis, all hub genes showed good prognostic value with an AUC ≥ 0.7 (Fig. 4C–L). Three hub genes (PTGS2, CBL, and RUNX2) showed excellent prognostic value with an AUC ≥ 0.9. The RUNX2 gene generated the highest AUC (AUC = 0.97, 95% CI 0.92–1.00) of all the hub genes (Table 4).

**Table 4:** Validation of hub genes by ROC analysis.

Gene	AUC	SE	Adj *P*-value	95% CI
PTGS2	0.900	0.072	< 0.001	0.749–1.000
LRRK2	0.810	0.092	0.004	0.621–0.998
ANXA5	0.857	0.089	0.001	0.683–1.000
IGF1R	0.890	0.059	< 0.001	0.775–1.000
MCL1	0.877	0.067	< 0.001	0.744–1.000
TIMP2	0.863	0.080	0.001	0.705–1.000
LYN	0.890	0.071	< 0.001	0.751–1.000
CD68	0.757	0.104	0.016	0.553–0.970
CBL	0.930	0.051	< 0.001	0.830–1.000
RUNX2	0.970	0.024	< 0.001	0.922–1.000

*AUC* area under the receiver operating characteristic curve, *SE* standard error, *CI* confidence interval.

### Drug-gene-disease interaction

**Figure 5:**
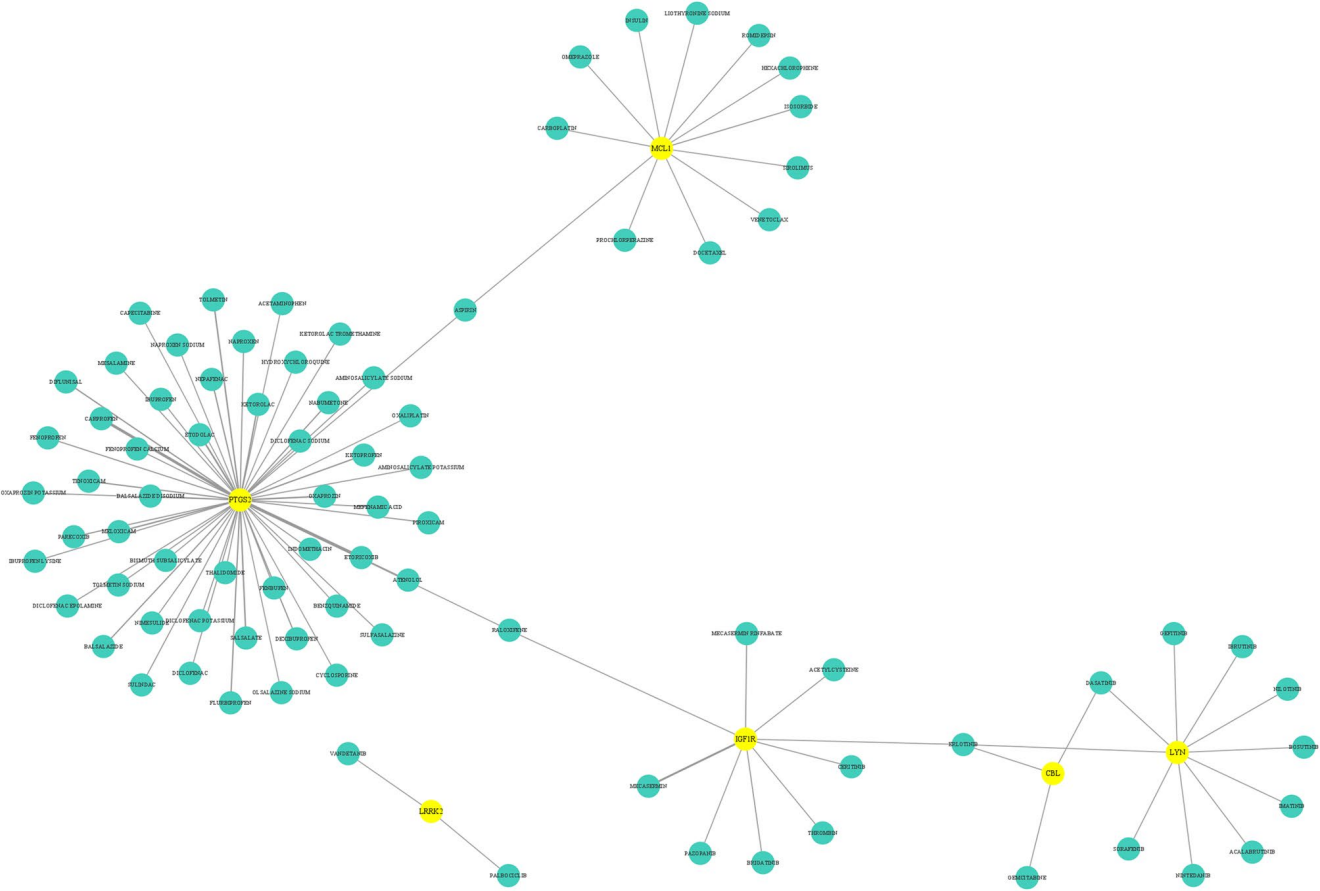
The drug-gene interactions network Constructed in Cytoscape. Yellow points represented genes and green points represented drugs. The thicker the connective line, the more the evidence.

A total of 85 FDA-approved drugs interacting with the 10 hub genes in ONFH were screened in the DGIdb (Table 5). The drug-gene interactions were visualized through Cytoscape (Fig. 5). PTGS2 was found to be the most frequently targeted gene by the majority of the drugs (53/85). Most interactive drugs exhibited inhibitory effects (54/85), with 52 being inhibitors and 2 being antagonists. Only 2 agonists were found, both of which were related to IGF1R. Two drugs (hydroxychloroquine and aspirin) were found to be protective against ONFH, while eight (cyclosporine, diclofenac, indomethacin, capecitabine, nilotinib, imatinib, sorafenib, and dasatinib) had the opposite effect. More details could be referred to in Supplementary Table S4.

**Table 5:** FDA-approved drugs interacting with hub genes.

Gene	Directionality	Numbers	Drugs
PTGS2	Inhibitory	46	Etoricoxib; Carprofen; Etodolac; Oxaprozin; Ketorolac; Salsalate; Tolmetin; Meloxicam; Ketoprofen; Flurbiprofen; Nepafenac; Diflunisal; Tenoxicam; Balsalazide; Dexibuprofen; Nabumetone; Naproxen; Parecoxib; Fenoprofen; Piroxicam; Mefenamic Acid; Fenoprofen Calcium; Aminosalicylate Potassium; Ibuprofen Lysine; Diclofenac Potassium; Diclofenac Epolamine; Oxaprozin Potassium; Sulindac; Aminosalicylate Sodium; Diclofenac; Ibuprofen; Indomethacin; Mesalamine; Ketorolac Tromethamine; Naproxen Sodium; Tolmetin Sodium; Nimesulide; Sulfasalazine; Balsalazide Disodium; Bismuth Subsalicylate; Olsalazine Sodium; Acetaminophen; Thalidomide; Diclofenac Sodium; Aspirin; Benzquinamide
	N.A	7	Fenbufen; Hydroxychloroquine; Raloxifene; Oxaliplatin; Capecitabine; Cyclosporine; AtenoloL
LRRK2	N.A	2	Palbociclib; Vandetanib
IGF1R	Activating	2	Mecasermin; Mecasermin rinfabate
	Inhibitory	2	Brigatinib; Ceritinib
	N.A	5	Raloxifene; Acetylcysteine; Thrombin; Erlotinib; Pazopanib
MCL1	Inhibitory	1	Venetoclax
	N.A	11	Isosorbide; Romidepsin; Aspirin; Prochlorperazine; Insulin; Liothyronine sodium; Sirolimus; Omeprazole; Hexachlorophene; Carboplatin; Docetaxel
LYN	Inhibitory	5	Nintedanib; Ibrutinib; Bosutinib; Acalabrutinib; Dasatinib
	N.A	5	Nilotinib; Erlotinib; Imatinib; Gefitinib; Sorafenib
CBL	N.A	3	Erlotinib; Dasatinib; Gemcitabine

N.A: not available in the database.

## Discussion

The pathogenesis of ONFH is still obscure. There are five mainstream hypotheses attempting to illustrate how the ONFH occurs, including the lipid metabolism disorder theory, the decreased osteogenesis potential of bone marrow mesenchymal stem cells (BMMSCs) theory, the insufficient blood supply theory, the inflammation and cell apoptosis theory, and the gene polymorphism and non-coding RNA theory^6^. In the past few years, growing attention has been paid to the regulatory mechanism of ONFH at the gene level. However, different gene expressions between blood and local tissues have not been noted in previous studies^11–14^. Our study is the first to describe this finding.

Our study has identified 10 hub genes. The first gene, PTGS2, also known as cyclooxygenase-2 (COX-2), is a key enzyme in the initiation of prostaglandin synthesis and inflammatory responses. The inflammation and cell apoptosis theory proposed that the main pathological process in ONFH was an increase in osteoblast and osteocyte apoptosis caused by inflammation^6^. But we have some unusual findings. Our analysis showed that PTGS2 is down-regulated in ONFH patients, as are some other typical inflammatory genes such as AKT, MAPK, and STAT. We found none of the inflammation-related signaling pathways, such as the TNF-α and the NF-κB signaling pathways, were significantly enriched when an adjusted *P*-value < 0.05 was considered statistically significant. Moreover, our investigation revealed that certain non-selective non-steroidal anti-inflammatory drugs (NSAIDs) had been reported to be associated with the development of ONFH, such as diclofenac^15^ and indomethacin^16^. These unexpected findings suggest that non-selective NSAIDs, commonly prescribed to alleviate pain in patients with ONFH, may pose medication risks that have not been given due attention by orthopedic surgeons^4^. Zheng et al.^17^ figured out that COX-2 enhanced osteogenic activity in rats. Zhang et al.^18^ reported that glucocorticoid induced ONFH via the COX-2-PGE-2 (prostaglandin E2)-HIF-1α (hypoxia-inducible factor-1α) axis. They found that dexamethasone repressed COX-2 expression, thereby reducing HIF-1α expression in femoral heads. The implantation of BMMSCs overexpressing HIF-1α into femoral heads of ONFH mice significantly reduced osteonecrotic areas and enhanced bone repair, thus largely preserving the structural integrity of femoral heads. Our study suggests that COX-2 may play an important role in the decreased osteogenesis potential of BMMSCs theory and serve as a protective factor in the process of ONFH. We recommend that orthopedic surgeons carefully use non-selective NSAIDs for analgesia in ONFH patients.

The third hub gene, ANXA5, is a kind of calcium-binding protein associated with osteoblasts differentiation and bone formation^19,20^. Genetos et al.^20^ reported a significant reduction in proliferation in pre-osteoblasts with ANXA5-knockdown, providing evidence that ANXA5 impact bone formation by regulating osteoprogenitor proliferation, differentiation, and cytokine responsiveness. On the other hand, ANXA5 functions as an anticoagulant protein that indirectly inhibits the thromboplastin-specific complex and plays a role in the blood coagulation cascade. Interestingly, we found that ANAX5 was significantly down-regulated in ONFH patients, and hydroxychloroquine, a potential therapeutic drug for ONFH, was strongly associated with ANXA5. Previous studies have elucidated that hydroxychloroquine could inhibit thrombosis by protecting the ANXA5 anticoagulant shield from disruption by antiphospholipid antibodies (aPLs)^21,22^. Our investigation revealed a close association between aPLs and the incidence of ONFH^23–27^, and a large amount of clinical evidence supports the protective effect of hydroxychloroquine against ONFH^27–31^. Long et al.^27^conducted an 11-year cohort study comprising 1158 patients to identify possible risk factors in Chinese systemic lupus erythematosus (SLE) patients. They found that the presence of aPLs was a risk factor for multiple joint necrosis (OR = 6.28, 95% CI 1.573–25.120; *p* = 0.009), and the administration of hydroxychloroquine was a protective factor for ONFH (OR = 0.547, 95% CI 0.320–0.937; *p* = 0.028). Hamza et al.^28^ conducted a cross-sectional case–control study to assess the risk factors for advanced ONFH. They found that a higher proportion of patients without symptomatic ONFH received hydroxychloroquine than patients with symptomatic ONFH (83.3% vs. 60%, *p* = 0.045). The authors concluded that the use of hydroxychloroquine had a protective effect on the development of ONFH, which was consistent with many other reports^29–31^. Although the hydroxychloroquine was initially discovered through the interaction with PTGS2 in this study, the subsequent literature mining results suggest a plausible drug-gene-disease correlation between ANXA5, hydroxychloroquine, and ONFH, which could help repurpose hydroxychloroquine.

IGF1R is a typical osteogenesis-related protein and plays a fundamental role in bone formation and angiogenesis. Several studies utilizing mouse or rabbit models have demonstrated that elevated local and/or systemic concentrations of IGF-1 confer advantageous outcomes in steroid-induced ONFH^32–37^. Xie et al.^38^ have reported that IGF-I and IGF-IR stimulate cell proliferation and impede cell apoptosis, which significantly suppress the development of ONFH. In this research, IGF1R is ranked fourth among all hub genes and is found to be primarily involved in signal transduction, negative regulation of apoptotic process, and protein binding function enrichment. Among the nine drugs found to interact with IGF1R, raloxifene has been verified to have a positive effect on the up-regulation of IGF1R expression and bone maintenance^39^. Mecasermin and mecasermin rinfabate have also been proven effective as IGF1R ligands in bone growth and maintenance^40–42^. We suppose that IGF1R is expected to be a valuable therapeutic target for ONFH.

The hub gene MCL1 encodes an anti-apoptotic protein that is required for the development of chronic myeloid leukemia (CML)^43–45^. The occlusion of microcirculation caused by the aggregation of leukemic cells is considered one of the causes of ONFH^43,46^. Unfortunately, no studies have yet proposed therapeutic strategies that would allow inhibition of this process. Through integrated bioinformatics analysis, we find that there is an interaction between aspirin and MCL1. Aspirin could decrease the MCL1 expression level independently of the NF-κB and MAPKs pathways, promoting human leukemia cells apoptosis^47,48^. Albers et al.^49^ conducted a prospective cohort study and discovered that aspirin has a therapeutic effect on ONFH during the 3.7-year follow-up. This newly discovered drug-gene-disease correlation in the current study suggests that aspirin may be able to partially prevent ONFH secondary to CML by suppressing MCL1, and indicates a novel use of the traditional drug.

The hub gene LYN is intimately correlated with MCL1. LYN has been shown to regulate the expression of MCL1 via Akt activity^50^. Through literature mining, we find that all validated chemotherapeutic agents targeting LYN, such as nilotinib^51^, imatinib^52^, sorafenib^53^, and dasatinib^54^, exhibit unfavorable effects on ONFH. The potential risk that such drugs may pose to ONFH should be treated cautiously.

The E3 ubiquitin ligases CBL plays a key role in bone formation and maintenance by regulating cellular proliferation and migration^55,56^. Our analysis demonstrates that CBL interacts with IGF1R and LYN. CBL exerts a considerable effect on a variety of osteoclast signaling pathways, including NF-κB, PI3K, and M-CSF receptors^57^. Simultaneously, CBL modulates osteoblast differentiation in mesenchymal cells through the ubiquitin–proteasome pathway^55^. We found that CBL was significantly underexpressed in patients with ONFH. Given the dual regulation of both osteoblast and osteoclast signals and the multi-gene interactions of CBL, our findings suggest CBL is a novel biomarker in ONFH.

RUNX2 is the last hub gene we identified. RUNX2 is a transcription factor that plays a critical role in osteoblast differentiation and skeletal morphogenesis. It is essential for osteoblast maturation as well as intramembranous and endochondral ossification, which are thought to be closely related to ONFH in the decreased osteogenesis potential of BMMSCs theory. Yang et al.^58^comfirmed that the genotypes of RUNX2 rs3763190 (G/A) were statistically associated with a higher ONFH risk. Our analysis shows that RUNX2 is down-regulated in ONFH and interacts with PTGS2, ANXA5, and TIMP2. Enhanced expression of RUNX2 elevates pro-osteogenic activity^59^. Li et al.^60^ reported that RUNX2 could directly regulate the downstream target VEGF to stimulate angiogenesis. A significant number of drugs have a regulatory effect on the RUNX2 gene. Our research identified cyclosporine as a drug that has a detrimental effect on ONFH. The drug-gene interaction reveals that cyclosporine A reduces RUNX2 expression through the calcineurin/NFAT pathway in various environments, thereby inhibiting osteoblastogenesis^61–64^. Cyclosporine has been demonstrated to be an independent risk factor for ONFH after kidney transplantation^65^. It is important to note that while several studies have reported a reduction in the incidence of ONFH with the use of cyclosporine, this effect is attributed to cyclosporine lowering the steroid dose^66–70^. When adjusted for this factor, cyclosporine itself increases the risk of ONFH^71^. These discoveries help further understand the possible mechanism of ONFH.

In addition to the pharmaceuticals identified in this study, those mentioned in the most recent expert consensus also warrant consideration, including bisphosphonates, anticoagulants, vasodilators, and lipid lowering agents^4^. Briefly, bisphosphonates and anticoagulants are considered to be helpful for early ONFH, and clinicians can consider using them as appropriate. The evidence to support the use of statins or vasodilators in the treatment of ONFH is very low and their use cannot be recommended^4,72–74^.

Bisphosphonates not only cause osteoclast apoptosis but also prevent osteocyte and osteoblast apoptosis^75–77^. We found that three hub genes (PTGS2, MCL-1, and RUNX2) may play a mediating role. Risedronate was reported to enhance bone formation via the up-regulation of COX-2 expression^78^. Zoledronic acid induces apoptosis in osteoclast precursors and mature osteoclast-like cells by triggering Mcl-1 down-regulation^79^. RANKL increases the level of Mcl-1 in osteoclasts and significantly attenuates the ability of both clodronate and alendronate to induce osteoclast apoptosis^80^. Various bisphosphonates have been shown to promote osteogenic differentiation by up-regulating the expression of RUNX2^81–84^. The use of bisphosphonates for treating ONFH is highly anticipated. Nevertheless, the clinical efficacy of bisphosphonates remains a topic of debate. While numerous reports indicate that bisphosphonates alleviate pain, enhance ambulation, and delay joint collapse in patients with ONFH^73,85–93^, there is also a substantial amount of negative evidence, particularly from high-quality meta-analyses of randomized control trials^94–98^. Serious side effects of bisphosphonates, such as atypical femoral fractures^99,100^ and osteonecrosis of the jaw^100–103^, are also of great concern. We recommend that caution be taken with the use of bisphosphonates to treat ONFH, as mentioned in guidelines, more high-quality random clinical trials are needed to prove its efficacy^3,4^.

The main mechanism of anticoagulant treatment for ONFH is the alleviation of hypercoagulability^104^. Hypercoagulability has been demonstrated to not only induce primary ONFH^3,104,105^, but also ONFH that is secondary to certain diseases, such as thrombophilia^106,107^ and antiphospholipid syndrome^23,108^. Anticoagulants have been proven to have significant protective effects on ONFH caused by different causes^105,109–114^. The results of literature mining suggest that anticoagulants may also possess other potential mechanisms for protecting against ONFH. Specifically, anticoagulant treatment has been found to have pro-osteogenic and pro-angiogenic effects, with the hub gene RUNX2 potentially playing a role in mediating this process^115,116^. These findings are conducive to further reveal the molecular mechanism of anticoagulant treatment of ONFH.

We do have some weak findings. CD68 is a macrophages-specific immunomarker that regulates osteoclast activity and is engaged in macrophage response and antigen presentation but does not participate in inflammation^117^. CD68 was previously found to be significantly elevated in synovial tissue near the necrotic femoral head^118,119^. For the first time, we discovered that CD68 is down-regulated in cartilage and peripheral blood. This unusual finding deserves more attention in future studies. LRRK2 is a functional protein kinase and guanosine diphosphatase/guanosine triphosphate-binding protein that is widely expressed in multiple tissues. Our analysis shows that LRRK2 ranks second among 10 hub genes and participates in almost all functional enrichment terms. However, a previous study demonstrated that the specific inhibition of LRRK2 does not result in changes in bone mass^120^. The precise role of LRRK2 in ONFH warrants additional investigation. TIMP2 is a kind of endogenous tissue inhibitor of matrix metalloproteases (MMPs). Although TIMP2 expression is associated with MMPs in bone remodeling, its effect on bone metabolism appears to be non-essential^121^. More studies are required to elucidate the mechanism.

There are some limitations in our research. Due to the limited data sources, we are unable to select expression profiling data from the same GPL platform, which may bring bias as a consequence of batch effects and biological differences. We have to set a threshold for GEO2R analysis as the adjusted *P*-value < 0.05 to meet the upper limit of the analysis tool. The strict inclusion criteria may lead to the loss of some DEGs. We will conduct more in-depth research in the future.

## Conclusions

In summary, a total of 159 DEGs and 85 interacting drugs were screened through integrated bioinformatics analysis and literature mining. PTGS2, LRRK2, ANXA5, IGF1R, MCL1, TIMP2, LYN, CD68, CBL, and RUNX2 were identified as 10 hub genes, which has considerable implications for future genetic research and related research fields of ONFH, especially genomics-driven drug development. We revealed that most drugs that showed a positive influence on ONFH had the effect of promoting osteogenesis and angiogenesis or inhibiting microcirculation embolism rather than being anti-inflammatory. Our findings help further understand the pathogenesis of ONFH, and guide drug development, and of more importance, provide new insights into the prevention and treatment of ONFH.

## Materials and methods

### Data obtaining and preprocessing

Two microarray datasets (GSE123568 and GSE74089) were downloaded from the GEO database. The inclusion criteria were set as follows: (1) the samples were obtained from humans; (2) the data meet gene expression profiling by array; (3) there are at least 3 samples in each group.

The GSE123568 datasets consisted of 30 cases and 10 controls and were based on the GPL15207 platform (Affymetrix Human Gene Expression Array). The GSE74089 datasets consisted of 4 cases and 4 controls and were based on the GPL13497 platform (Agilent-026652 Whole Human Genome Microarray 4 × 44K v2). Details were listed in Table 1. After filtering out the duplicate probes or probes without corresponding gene symbols, the rest of the probes were transformed into the homologous gene symbol according to the platform’s annotation information.

### Identification of DEGs

GEO2R is an interactive web tool that can compare and analyze the GEO series^122^. Included datasets were analyzed by GEO2R to identify DEGs, and then the results were downloaded in tsv format. DEGs were evaluated according to the cutoff criteria of an adjusted *P*-value < 0.05 and |log2FC (fold change)|> 1.0. Subsequently, the FunRich tool (version 3.1.3) was used to examine the intersection of DEGs and construct the Venn diagram^123^. A visual hierarchical cluster analysis was conducted through the limma package in software R (version 4.1.1) to present the volcano plot. Additionally, an integrative toolkit, TBtools, was used to draw the heatmap of DEGs^124^.

### GO and KEGG enrichment analysis of DEGs

GO functional analysis and KEGG pathway analysis were carried out by the Database for Annotation, Visualization, and Integrated Discovery (DAVID) to identify enriched biological themes and discover enriched functional-related gene groups^125–127^. The top 10 or all (if less than 10) significant items of the biological process (BP), cellular component (CC), molecular function (MF) categories, and KEGG pathways were picked up for further validation and presented in the form of bubble maps. These bubble plots were drawn by the statistical software R (version 4.1.1), using the ggplot2 R package. And a *P*-value < 0.05 were considered statistically significant.

### PPI network construction and visualization

A protein–protein interaction (PPI) network of the identified DEGs with an interaction score > 0.4 was constructed by the Search Tool for the Retrieval of Interacting Genes/Proteins (STRING). The DEGs and their related genes were presented with at least 2 interactions. Subsequently, the functional modules of the PPI network were screened and visualized using the Cytoscape software (version 3.8.2)^128^. The parameters of Cytoscape plugin Molecular Complex Detection (MCODE) were as follows: degree cutoff = 2, node score cutoff = 0.2, k-score = 2, and max.depth = 100^129^. GO functional analysis and KEGG pathway analysis were performed for every module by DAVID.

### Target gene screening

A topology analysis was performed, and based on the highest score of the Degree, BottleNeck, and MCC algorithms, ten genes were selected as the target hub genes^130,131^. The PPI network of the hub genes with each other was constructed using the Cytoscape plugin cytoHubba^130^. In the meantime, the interaction network of the hub genes and their related genes was established in FunRich software. The criteria of the network included a confidence score ≥ 0.4 and a minimum number of interactions ≥ 1. Meanwhile, KEGG pathway analysis was performed in STRING. Moreover, the area under the receiver operating characteristic (ROC) curve (AUC) was analyzed for validation of the hub genes.

### Therapeutic drugs identification

The Drug-Gene Interaction Database (DGIdb) is a large comprehensive database for searching the existing compendia of known or potential drug-gene interactions against lists of genes. The 10 hub genes served as promising targets in the search for therapeutic drugs through the DGIdb . In this study, drugs that were approved by the Food and Drug Administration (FDA) would be included. All drugs were cross-validated by integrated literature mining. We searched for (“drug name”[MeSH Terms] OR “ drug name”[All Fields]) AND ((“femur head”[MeSH Terms] OR (“femur”[All Fields] AND “head”[All Fields]) OR “femur head”[All Fields] OR (“femoral”[All Fields] AND “head”[All Fields]) OR “femoral head”[All Fields]) AND (“necrose”[All Fields] OR “necrosed”[All Fields] OR “necrosis”[All Fields] OR “necrosing”[All Fields] OR “necrosis”[MeSH Terms] OR “necrosis”[All Fields] OR “necroses”[All Fields]) OR (“osteonecrosis”[MeSH Terms] OR “osteonecrosis”[All Fields] OR “osteonecrosis”[All Fields]) OR (“aseptic”[All Fields] AND “necrosis”[All Fields]) OR (“avascular”[All Fields] AND “necrosis”[All Fields])) in Web of Science and PubMed. Then we carefully read and extracted the useful information from each literature to determine the effectiveness (or lack thereof) of drugs. We identified the possible therapeutic drugs according to the drug–gene–disease triangulation model proposed by Sun et al.^10^. The Cytoscape was used to construct the drug-hub gene interaction network. The parameters were set as follows: source = drugs, target = genes, edge attribute = interaction score, edge width = continuous mapping [range, 1.0–3.0].

## Supplementary Information

Supplementary Information.

## Data Availability

The datasets generated and/or analysed for this study can be found in the GEO repository. GSE123568 is available at: https://www.ncbi.nlm.nih.gov/geo/query/acc.cgi?acc=GSE123568, GSE74089 is available at: https://www.ncbi.nlm.nih.gov/geo/query/acc.cgi?acc=GSE74089.
