# Supplementary material for: Identification of hub genes and therapeutic drugs in osteonecrosis of the femoral head through integrated bioinformatics analysis and literature mining
Source: Sci Rep. 2023 Jul 24;13:11972. doi: 10.1038/s41598-023-39258-4 (PMC10366127; doi:10.1038/s41598-023-39258-4)
Supplement: Supplementary file 1 — Supplementary Information. [file 41598_2023_39258_MOESM1_ESM.pdf]

Identification of hub genes and therapeutic drugs in osteonecrosis of the femoral head through integrated bioinformatics analysis and literature mining

Lan Tang[1,2†], Bin Li[1,2†], Qiuming Su[3†], Xi Chen[1,4], Rongxin He[1,2*]

[†] Lan Tang, Bin Li, and Qiuming Su contributed equally to this work and share first authorship.

[1] Department of Orthopedic, The Second Affiliated Hospital, Zhejiang University School of Medicine, Hangzhou City, Zhejiang Province, China;

[2] Key Laboratory of Motor System Disease Research and Precision Therapy of Zhejiang Province, Hangzhou City, Zhejiang Province, China;

[3] Department of Hepatopancreatobiliary Surgery, The First People's Hospital of Kunming, Calmette Hospital, Kunming City, Yunnan Province, China;

[4] Department of Epidemiology and Statistics, School of Public Health, Medical College, Zhejiang University, Hangzhou City, Zhejiang Province, China.

Corresponding author: Rongxin He, Department of Orthopedic, The Second Affiliated Hospital, Zhejiang University School of Medicine, Hangzhou City, Zhejiang Province, PR China, #88 Jiefang Road, Hangzhou 310001, China. E-mail address: herongxin@zju.edu.cn. Tel: +86-571-87783530; Fax: +86-571-87022776. 0000-0002-5937-3878 (ORCID)

Supplementary Table S1: The significant terms of GO enrichment analysis results (determined by *P*-Value < 0.05)

Category	Term ID	Term	Count	Gene Ratio	*P*-Value	Adj *P*-Value	Genes
**KEGG_PATHWAY**	hsa05152:	Tuberculosis	6	3.80	0.024	0.964	IFNGR2, LAMP2, CD14, NOD2, BID, CAMK2G
	hsa04142:	Lysosome	5	3.16	0.026	0.972	LAMP2, PSAP, PPT1, NAGA, CD68
	hsa04966:	Collecting duct acid secretion	3	1.90	0.026	0.972	ATP6V1A, ATP6V1B2, SLC4A1
	hsa04064:	NF-kappa B signaling pathway	4	2.53	0.047	0.999	LYN, CD14, CFLAR, PTGS2
**GOTERM_BP_DIRECT**	GO:0007165	signal transduction	18	11.39	0.018	1.000	LYN, ARHGAP9, ANXA5, ARRDC3, ARHGAP27, GNG11, ANK1, CXCL5, RAP1GAP, IGF1R, THBD, CEACAM6, NAMPT, PPFIA1, PDE4B, PKN2, RIN2, SKAP2
	GO:0043547	positive regulation of GTPase activity	12	7.59	0.008	1.000	ARHGAP9, RGS2, DOCK8, LRRK2, DENND3, ASAP1, ARHGAP27, SMAP2, CAMK2G, RIN2, SPTB, RAP1GAP
	GO:0035556	intracellular signal transduction	11	6.96	0.002	0.914	SRPK2, RPS6KA5, ANP32A, DUSP1, WNK1, LRRK2, PKN2, NOD2, SGK1, TREM1, GPR155
	GO:0043066	negative regulation of apoptotic process	10	6.33	0.015	1.000	CITED2, DUSP1, ANXA5, PPT1, FHL2, CFLAR, CBL, IGF1R, MCL1, TXNDC5
	GO:0042981	regulation of apoptotic process	8	5.06	0.002	0.914	HIP1, ANP32A, DUSP1, CFLAR, NOD2, BID, SGK1, MCL1
	GO:0010628	positive regulation of gene expression	7	4.43	0.023	1.000	SRPK2, PID1, CITED2, MSN, KLF4, CD46, QKI

Continued

Category	Term ID	Term	Count	Gene Ratio	*P*-Value	Adj *P*-Value	Genes
	GO:0008286	insulin receptor signaling pathway	6	3.80	0.001	0.435	ATP6V1A, NAMPT, ATP6V1B2, PDK4, RHOQ, IGF1R
	GO:0050900	leukocyte migration	6	3.80	0.004	0.985	LYN, THBD, CEACAM6, PDE4B, MSN, TREM1
	GO:0008360	regulation of cell shape	6	3.80	0.007	0.999	WIPF1, EPB41L3, MSN, PLEKHO1, MYH10, RHOQ
	GO:0007010	cytoskeleton organization	5	3.16	0.047	1.000	SVIL, HIP1, MSN, SPTB, ANK1
**GOTERM_CC_DIRECT**	GO:0005737	cytoplasm	63	39.87	0.002	0.281	HIP1, PID1, CITED2, LRRK2, BACH1, LYST, RGS2, RPS6KA5, BASP1, NAMPT, ENSA, PPFIA1, LRRFIP1, RNF111, DUSP1, ARG1, ANXA5, ANK1, SPTB, NBPF1, RUNX2, RNF144B, C1ORF116, SPOPL, WDFY3, PLEKHO1, MPPE1, SGK1, RIN2, SKAP2, KCNE3, ANP32A, FAM129A, ABHD5, NOD2, CBL, PTGS2, ZFP36L2, PSAP, EPB41L3, NHS, BID, RNF130, MYH10, MCL1, EGLN1, SRPK2, SPAG9, LYN, SVIL, NAGA, MSN, CFLAR, ARHGAP27, KLF4, CPPED1, QKI, PTPRE, IMPDH1, WNK1, PKN2, TACC1, SMAP2

Continued

Category	Term ID	Term	Count	Gene Ratio	*P*-Value	Adj *P*-Value	Genes
	GO:0005886	plasma membrane	52	32.91	0.002	0.362	LRRK2, SLC4A1, TREM1, IGF1R, THBD, GLIPR1, RGS2, BASP1, LAMP2, LRRFIP1, IL13RA1, WLS, RAB2B, IFNGR2, ARRDC3, ATP11A, ANK1, RHOB, IL17RA, CEACAM6, ATP6V1B2, RELL1, SGK1, CD46, RHOQ, SKAP2, ATP6V1A, KCNE3, ABHD3, FAM129A, NOD2, CBL, FCHO2, EPB41L3, S1PR3, CD14, MYH10, CAMK2G, LYN, SLC14A1, SVIL, KCNJ15, MSN, GNG11, CPPED1, SULF2, VMP1, SLC6A6, PTPRE, PKN2, TGFBI, CD68
	GO:0005829	cytosol	45	28.48	0.001	0.227	ATP6V1A, ARHGAP9, PFKFB4, WIPF1, DOCK8, LRRK2, ASAP1, ABHD5, NOD2, CBL, BACH1, RAP1GAP, ZFP36L2, GSPT1, RGS2, ANPEP, NAMPT, PDE4B, PPFIA1, RICTOR, TK1, LRRFIP1, BID, MYH10, CAMK2G, MCL1, SPAG9, LYN, EGLN1, ARG1, CFLAR, ANK1, SPTB, RHOB, RNF144B, IMPDH1, WNK1, PELI1, ATP6V1B2, PPT1, PKN2, CEP63, SGK1, RHOQ, SKAP2

Continued

Category	Term ID	Term	Count	Gene Ratio	*P*-Value	Adj *P*-Value	Genes
	GO:0070062	extracellular exosome	38	24.05	0.004	0.561	ATP6V1A, FAM49B, MTMR11, LRRK2, FAM129A, SLC4A1, NID1, BASP1, ANPEP, LAMP2, NAMPT, PSAP, TIMP2, CD14, BID, GPR155, CTBS, MYH10, TXNDC5, WLS, SPAG9, LYN, RAB2B, ARG1, FUCA2, ANXA5, NAGA, MSN, CPPED1, RHOB, C1ORF116, ATP6V1B2, PPT1, TGFBI, KCTD12, CD46, SMPDL3A, RHOQ
	GO:0016020	membrane	28	17.72	0.031	0.999	FAM49B, HIP1, KCNE3, DOCK8, FAM129A, MOSPD2, ASAP1, RAP1GAP, IGF1R, OGFRL1, GLIPR1, LAMP2, PDE4B, BID, CAMK2G, MCL1, IKBIP, OSBPL8, ANXA5, KCNJ15, ATP11A, ARHGAP27, VMP1, VCAN, WNK1, PPT1, TACC1, CD68
	GO:0005615	extracellular space	20	12.66	0.020	0.989	ARG1, LRRK2, FUCA2, MSN, CXCL5, SULF2, THBD, VCAN, CEACAM6, ANPEP, DMXL2, LAMP2, NAMPT, PSAP, PPT1, TIMP2, CD14, TGFBI, SMPDL3A, CTBS
	GO:0045121	membrane raft	8	5.06	0.002	0.344	LYN, KCNE3, PPT1, EFHD2, CD14, CFLAR, CBL, RHOQ

Continued

Category	Term ID	Term	Count	Gene Ratio	*P*-Value	Adj *P*-Value	Genes
	GO:0005765	lysosomal membrane	8	5.06	0.009	0.863	ATP6V1A, ANPEP, LAMP2, PSAP, ATP6V1B2, ATP11A, CD68, SLC15A4
	GO:0005925	focal adhesion	8	5.06	0.050	1.000	SVIL, ANXA5, FHL2, PPFIA1, NHS, MSN, CD46, RHOB
	GO:0005764	lysosome	7	4.43	0.013	0.941	LRRK2, LAMP2, ARRDC3, PSAP, PPT1, NAGA, CTBS
**GOTERM_MF_DIRECT**	GO:0005515	protein binding	101	63.92	< 0.001	0.001	FAM49B, PID1, WIPF1, JMJD1C, SLC4A1, BACH1, IGF1R, RGS2, RPS6KA5, BASP1, LAMP2, NAMPT, PPFIA1, RNF111, IL13RA1, WLS, RAB2B, ARRDC3, ATP11A, ANK1, RUNX2, C1ORF116, TFR2, WDFY3, MPPE1, SKAP2, TSHZ3, KCNE3, ANP32A, RAP1GAP, GSPT1, ZFP36L2, PSAP, EPB41L3, CD14, MYH10, MCL1, TXNDC5, SRPK2, LYN, FUCA2, MSN, QKI, PTPRE, MAFB, WNK1, PPT1, TGFBI, MXD1, ARHGAP9, HIP1, CITED2, DOCK8, LRRK2, FHL2, LYST, THBD, ENSA, TIMP2, TK1, LRRFIP1, IKBIP, DUSP1, ANXA5, TET2, SPTB, RHOB, IL17RA, RNF144B, VCAN, CEACAM6, ATP6V1B2, PELI1, SPOPL, PLEKHO1, CEP63, SGK1, CD46, SMPDL3A, FAM129A, ASAP1, NOD2, CBL, PTGS2, FCHO2, RICTOR, S1PR3, BID, MPZL3, CAMK2G, EGLN1, SPAG9, SVIL, FAM46A, KCNJ15, CFLAR, KLF4, VMP1, PKN2, TACC1, SMAP2

Continued

Category	Term ID	Term	Count	Gene Ratio	*P*-Value	Adj *P*-Value	Genes
	GO:0005096	GTPase activator activity	8	5.06	0.009	0.955	ARHGAP9, RGS2, LRRK2, ASAP1, ARHGAP27, SMAP2, RIN2, RAP1GAP
	GO:0003924	GTPase activity	7	4.43	0.014	0.992	RAB31, LRRK2, GNG11, RAP1GAP, GSPT1, RHOQ, RHOB
	GO:0003779	actin binding	7	4.43	0.029	1.000	WIPF1, LRRK2, EPB41L3, MSN, NOD2, MYH10, SPTB
	GO:0005200	structural constituent of cytoskeleton	5	3.16	0.014	0.991	HIP1, EPB41L3, MSN, SPTB, ANK1
	GO:0017124	SH3 domain binding	5	3.16	0.018	0.998	LYN, WIPF1, ARHGAP27, CBL, QKI
	GO:0051015	actin filament binding	5	3.16	0.025	1.000	SVIL, HIP1, WIPF1, MYH10, SPTB

Supplementary Table S2: The interaction of the hub genes and their related genes discovered in FunRich database

Gene	Interacting partners
LYN	CTLA4; RASA1; ACTB; UNC119; RPS6KB2; CASP3; ITGA4; DOK3; GRIA3; LCP2; TYK2; CD19; PTK2; CSF2RB; TRAT1; CD22; CD79B; MATK; SPHK1; EGFR; FCGR2A; KIT; BANK1; CD72; CD24; PAG1; PRAM1; PRKCD; DOK2; CD36; CDK2; MUC1; CASP7; EPOR; CDK1; CSNK2B; SKAP1; TRPV4; LIME1; CDKN1B; PILRB; GP6; KHDRBS1; FCAR; SKAP2; PECAM1; SH2B2; GAB3; HNRNPK; TEC; SYK; UBB; FASLG; RPL10; SPHK2; PPP1R8; SLC4A1; DLG4; DOK1; HCLS1; CRKL; PIK3CG; PPP1R15A; DAPP1; PTPN6; ADAM15; PRKDC; BTK; CSF3R; CBLC; GAB2; CDK4; FCGR2B; CSK; JAK2; NEDD9; CSF1R; EVL; PTPRC; PDE4A; TRIP10; PAK2; MS4A2; SHC1; RPS6KB1; CASP9; PTK2B; PLCG1; ITPR1; CBL; IL2RB; ITGB1; IL7R; BCAR1; INPP5D; PRKCQ; CSF2RA; MAPK3; MAP4K1; NPHS1; MME; PLCG2; FCER1G; NMT1; CD79A; SNCA; CHST15; SRC; MS4A1; MAP3K3; FOLR1; RGS16
CBL	LYN; YWHAB; PIK3R1; MET; SH3KBP1; SPRY2; CRKL; VAV1; SPTA1; PLCG1; YWHAQ; FNBP1; PTPN6; ARHGEF7; FYN; PDGFRA; SLA2; TYK2; SLA; SH3BP2; BCR; MST1R; UBE2L3; EGFR; LCP2; SYK; PTK2B; PTPN22; VAV2; FGR; MYO1C; TRAF6; PTPRB; EPHB6; GRB2; CD2AP; CSK; EPHA2; PIK3CA; BLK; ITK; KHDRBS1; SORBS2; NCK1; HCK; GAPVD1; KDR; ABL1; CSF1R; TNFRSF11A; PTPRO; UBA1; IGF1R; SH3GL2; CRK; UBE2D2; PTPRC; ITSN1; EIF5B; LAT; PRKCA; SRC; UBC; KRT18; SPRY4; LCK; EPOR; ITCH; PIK3R2; CD19; BTK; NOTCH1; CD5; SHC1; LTK; TRIM21; TLN1; OSTF1; CDKL2; SH2B2; YWHAZ; PTPN11; SORBS1; UBE2D1; UBE2G1; F2RL1; INPPL1; ZAP70; PIK3CB; PTPRJ; PTPRK; FRS2; UBE2M; PDGFRB; CD40; SCN5A; STAT5A; INSR; KIT; FYB; LAT2; ASAP1; FLOT1; AXL; PXN; RET; SH3GL3; CAPN1; STAT5B; PTPRG; CSF1; UBE2G2; YWHAG; UBASH3B

Supplementary Table S3: KEGG analysis results of the hub genes and their related genes				
**KEGG pathways**	**Description**	**Count in network**	**Strength**	**FDR**
hsa01521	EGFR tyrosine kinase inhibitor resistance	32 of 78	1.36	<0.001
hsa04215	Apoptosis - multiple species	12 of 30	1.35	<0.001
hsa05220	Chronic myeloid leukemia	28 of 75	1.32	<0.001
hsa04930	Type II diabetes mellitus	17 of 46	1.31	<0.001
hsa04664	Fc epsilon RI signaling pathway	24 of 66	1.31	<0.001
hsa04012	ErbB signaling pathway	30 of 83	1.3	<0.001
hsa04662	B cell receptor signaling pathway	28 of 78	1.3	<0.001
hsa04917	Prolactin signaling pathway	24 of 69	1.29	<0.001
hsa05223	Non-small cell lung cancer	24 of 68	1.29	<0.001
hsa05210	Colorectal cancer	29 of 82	1.29	<0.001
hsa04666	Fc gamma R-mediated phagocytosis	30 of 90	1.27	<0.001
hsa05213	Endometrial cancer	19 of 57	1.27	<0.001
hsa04960	Aldosterone-regulated sodium reabsorption	12 of 37	1.26	<0.001
hsa05214	Glioma	23 of 72	1.25	<0.001
hsa04370	VEGF signaling pathway	18 of 57	1.24	<0.001
hsa05212	Pancreatic cancer	23 of 73	1.24	<0.001
hsa01522	Endocrine resistance	30 of 95	1.24	<0.001
hsa04935	Growth hormone synthesis, secretion and action	35 of 118	1.22	<0.001
hsa04660	T cell receptor signaling pathway	29 of 101	1.2	<0.001
hsa01524	Platinum drug resistance	20 of 70	1.2	<0.001
hsa05235	PD-L1 expression and PD-1 checkpoint pathway in cancer	25 of 88	1.2	<0.001
hsa04520	Adherens junction	19 of 67	1.2	<0.001
hsa04722	Neurotrophin signaling pathway	32 of 114	1.19	<0.001

Supplementary Table S4: Drug-gene-disease interaction of the 10 hub genes

Gene	Drug	Interaction	Sources	Drug-gene interaction (PMID)	Drug-disease interaction (PMID/DOI)
PTGS2	Ibuprofen	Inhibitor	TDG; CBD; TEND; GTP; PharmGKB; TTD	25502615; 15481334; 12852704; 11752352; 15556152; 15494133; 16678543; 14510637; 19602986	-
PTGS2	Flurbiprofen	Inhibitor	DTC; TDG; CBD; TEND; GTP; TTD	10091674; 10977131; 10773011; 11006278; 11405284; 17562170; 19066416	-
PTGS2	Balsalazide disodium	Inhibitor	CBD	-	-
PTGS2	Ketorolac tromethamine	Inhibitor	CBD	-	-
PTGS2	Benzquinamide	Inhibitor	GTP	-	-
PTGS2	Hydroxychloroquine	-	NCI	14963695	10.1016/j.ejr.2018.12.007♥; 21390576♦; 34082592♥; 32968109♥; 35236014♥; 16269429♥; 25300729♠
PTGS2	Fenoprofen calcium	Inhibitor	CBD	-	-
PTGS2	Aminosalicylate potassium	Inhibitor	CBD	-	-
PTGS2	Piroxicam	Inhibitor	TDG; CBD; GTP	11952155; 11785774; 11153163; 11752352; 15464832; 10381057	-
PTGS2	Bismuth subsalicylate	Inhibitor	CBD	-	-
PTGS2	Cyclosporine	-	NCI	10999939; 25124319	22882914♦; 17068716♣; 16968732♣; 29868874♠; 8377759♠; 3312208♥; 9850453♥; 3063055♥; 17524885♥; 8817751♥10.3109/17453678809149417♥
PTGS2	Diclofenac	Inhibitor	TDG; CBD; TEND; GTP; PharmGKB	12705061; 12925531; 12588370; 12852704; 12534640; 19602986	3453991♠; 26435236♣

Continued

Gene	Drug	Interaction	Sources	Drug-gene interaction (PMID)	Drug-disease interaction (PMID)
PTGS2	Nepafenac	Inhibitor	TDG; CBD; TEND	10850857	-
PTGS2	Ibuprofen lysine	Inhibitor	CBD	-	-
PTGS2	Naproxen sodium	Inhibitor	CBD	-	-
PTGS2	Diclofenac potassium	Inhibitor	CBD	-	-
PTGS2	Acetaminophen	Inhibitor	TDG; CBD; TEND; GTP	17884974; 17322116; 17175104; 11752352	-
PTGS2	Diclofenac sodium	Inhibitor	CBD	-	-
PTGS2	Indomethacin	Inhibitor	DTC; CBD; TEND; GTP; PharmGKB; TTD	15668944; 24697244; 15730717; 20684598; 15831440; 22818041; 15770365; 10381057; 15667901; 19602986	24917211[♦]; 1762005[♦]; 7281338[♠]; 10964124[♠]; 1013584[♣]
PTGS2	Diclofenac epolamine	Inhibitor	CBD	-	-
PTGS2	Parecoxib	Inhibitor	TDG	10794682	-
PTGS2	Ketorolac	Inhibitor	TDG; TEND; GTP	11695255; 12446609; 14568028; 14568029; 15102535; 12110513	-
PTGS2	Nimesulide	Inhibitor	GTP; PharmGKB	16846549; 11752352	-
PTGS2	Diflunisal	Inhibitor	TDG; CBD; TEND; TTD	11315375; 8737748; 12852483; 11673972; 10825891; 11752352	-
PTGS2	Balsalazide	Inhibitor	TDG	17981262; 12950415; 17139284; 17016423; 19743890	-
PTGS2	Thalidomide	Antagonist	TDG	12710892; 15446566; 15982930; 21507989; 15598423; 15892618	15955903[♣]

Continued

Gene	Drug	Interaction	Sources	Drug-gene interaction (PMID)	Drug-disease interaction (PMID)
PTGS2	Oxaliplatin	-	PharmGKB	19219602	-
PTGS2	Oxaprozin potassium	Inhibitor	CBD	-	-
PTGS2	Mesalamine	Inhibitor	TDG; CBD; TEND	16855178; 12463455; 14742690; 12208114; 9256165	-
PTGS2	Salsalate	Inhibitor	TDG; TEND	9711054; 10452868; 16808554; 12901032	-
PTGS2	Ketoprofen	Inhibitor	TDG; CBD; TEND; GTP; TTD	14513718; 11729362; 11752352; 11814865; 15198222; 10895904	-
PTGS2	Nabumetone	Inhibitor	TDG; CBD; TEND; TTD	11304699; 12047490; 11153163; 11752352; 15456329; 7615202	-
PTGS2	Fenbufen	-	TTD	-	-
PTGS2	Carprofen	Inhibitor	TDG; CBD; TEND; GTP; TTD	15939622; 11752352; 17181139; 11703020; 15198222; 10895904	-
PTGS2	Sulindac	Inhibitor	TDG; CBD; TEND; GTP	11118042; 10372826; 10657949; 10485483; 11078056	-
PTGS2	Naproxen	Inhibitor	TDG; CBD; TEND; GTP; PharmGKB; TTD	17604186; 17612049; 17607546; 11752352; 17521299; 17532719; 19602986; 30247840	-
PTGS2	Fenoprofen	Inhibitor	TDG; TEND	17050798	-
PTGS2	Mefenamic acid	Inhibitor	CBD	10393680; 15792781; 9626023; 17150210; 7832763; 11752352	-
PTGS2	Aminosalicylate sodium	Inhibitor	CBD	-	-

Continued

Gene	Drug	Interaction	Sources	Drug-gene interaction (PMID)	Drug-disease interaction (PMID)
PTGS2	Sulfasalazine	Inhibitor	TDG; CBD	16855178; 12463455; 14742690; 12208114; 9256165	-
PTGS2	Etoricoxib	Inhibitor	TDG; CBD; TEND; GTP; PharmGKB; TTD	17573128; 17164136; 17691997; 11752352; 17139284; 17016423; 19602986	-
PTGS2	Tolmetin	Inhibitor	TDG; TEND; TTD	14613550; 10465690; 9253954; 11752352; 17391279; 15458776	-
PTGS2	Olsalazine sodium	Inhibitor	CBD	-	-
PTGS2	Dexibuprofen	Inhibitor	DTC	20143779; 28009827	-
PTGS2	Raloxifene	-	NCI	15243281	-
PTGS2	Tolmetin sodium	Inhibitor	CBD	-	-
PTGS2	Meloxicam	Inhibitor	TDG; CBD; TEND; GTP; PharmGKB; TTD	10567199; 16197363; 10220944; 11752352; 10381787; 10340919; 10381057; 30774225; 19602986	-
PTGS2	Tenoxicam	Inhibitor	TDG; TEND; TTD	11563332; 16245223; 9152412; 11752352; 15943176; 15756931	-
PTGS2	Etodolac	Inhibitor	TDG; CBD; TEND; GTP; PharmGKB; TTD	12824918; 11009046; 11275997; 11752352; 10594327; 15198222; 19602986	-
PTGS2	Aspirin	Inhibitor	CBD; TEND; CIViC; GTP; PharmGKB	17522398	26011853♥; 11704199♥; 346279♥; 23041599♥
PTGS2	Capecitabine	-	PharmGKB	19219602	16969993♠
PTGS2	Atenolol	-	PharmGKB	-	-

Continued

Gene	Drug	Interaction	Sources	Drug-gene interaction (PMID)	Drug-disease interaction (PMID)
PTGS2	Oxaprozin	Inhibitor	TDG; CBD; TEND; GTP	19338579; 9831331; 9650852; 19952416; 12065695; 17024689	-
LRRK2	Vandetanib	-	DTC	-	-
LRRK2	Palbociclib	-	DTC	-	-
IGF1R	Mecasermin	Agonist	TDG; CBD; TEND; GTP; TTD	19198769	-
IGF1R	Brigatinib	Inhibitor	GTP	23239810; 28597393	-
IGF1R	Mecasermin rinfabate	Agonist	CBD	-	-
IGF1R	Ceritinib	Inhibitor	DTC; GTP	23837797	-
IGF1R	Erlotinib	-	CGI	20716637	-
IGF1R	Pazopanib	-	DTC	-	-
IGF1R	Raloxifene	-	NCI	14533013	-
IGF1R	Acetylcysteine	-	NCI	12485928	31115574[♦]
IGF1R	Thrombin	-	NCI	7499260; 11375274; 8550825; 8579617; 31694517	10.36468/pharmaceutical-sciences.spl.371[♦]; 18695356[♦]; 36684312[♣]
MCL1	Hexachlorophene	-	DTC	-	-
MCL1	Carboplatin	-	NCI	10081494	12665845[♦]
MCL1	Isosorbide	-	TDG	18084610	-
MCL1	Romidepsin	-	NCI	15059137	-
MCL1	Omeprazole	-	DTC	-	-
MCL1	Prochlorperazine	-	DTC	-	-
MCL1	Liothyronine sodium	-	DTC	-	8376558[♦]; 16996569[♦]
MCL1	Sirolimus	-	NCI	17010674	22882914[♦]; 31232079[♦]
MCL1	Venetoclax	Antagonist	GTP	-	-

Continued

Gene	Drug	Interaction	Sources	Drug-gene interaction (PMID)	Drug-disease interaction (PMID)
MCL1	Insulin	-	NCI	11527158	9005061♦
MCL1	Aspirin	-	NCI	12941295	26011853♥ ; 11704199♥ ; 346279♥; 23041599♥
MCL1	Docetaxel	-	NCI	16969094	27019628♦
LYN	Ibrutinib	Inhibitor	DTC; GTP	25222877	-
LYN	Bosutinib	Inhibitor	CBD; GTP	-	-
LYN	Gefitinib	-	DTC	-	-
LYN	Acalabrutinib	Inhibitor	GTP	-	-
LYN	Nilotinib	-	CIViC	18191450	29928099♠
LYN	Nintedanib	Inhibitor	TALC	18559524; 31016670	-
LYN	Erlotinib	-	DTC	-	-
LYN	Imatinib	-	DTC; CIViC	18191450	24383853♠
LYN	Sorafenib	-	DTC	-	19884542♠
LYN	Dasatinib	Inhibitor	CBD	18180381	34866312♣; 26279632♠
CBL	Erlotinib	-	JAX-CKB	25348515	-
CBL	Gemcitabine	-	JAX-CKB	25348515	-
CBL	Dasatinib	-	CGI	23696637	34866312♣; 26279632♠
RUNX2	Cyclosporine	-	-	16286645; 20554534; 31238868; 28219134	29868874♠; 8377759♠
ANXA5	Hydroxychloroquine	-	-	17053060; 19965621	10.1016/j.ejr.2018.12.007♥; 34082592♥; 32968109♥; 35236014♥; 16269429♥

TDG: The Druggable Genome Dataset; CBD: The ChEMBL Bioactivity Database; TEND: Trends in the exploitation of novel drug targets; GTP: Guide To Pharmacology; PharmGKB: The Pharmacogenomics Knowledgebase; TTD: Therapeutic Target Database; DTC: Drug Target Commons; NCI: National Cancer Institute Cancer Gene Index; CGI: Cancer Genome Interpreter; CIViC: Clinical Interpretation of Variants in Cancer; JAX-CKB: The Jackson Laboratory Clinical Knowledgebase; ♥Positive effect; ♠Negative effect; ♣Neutral effect; ♦Not sure

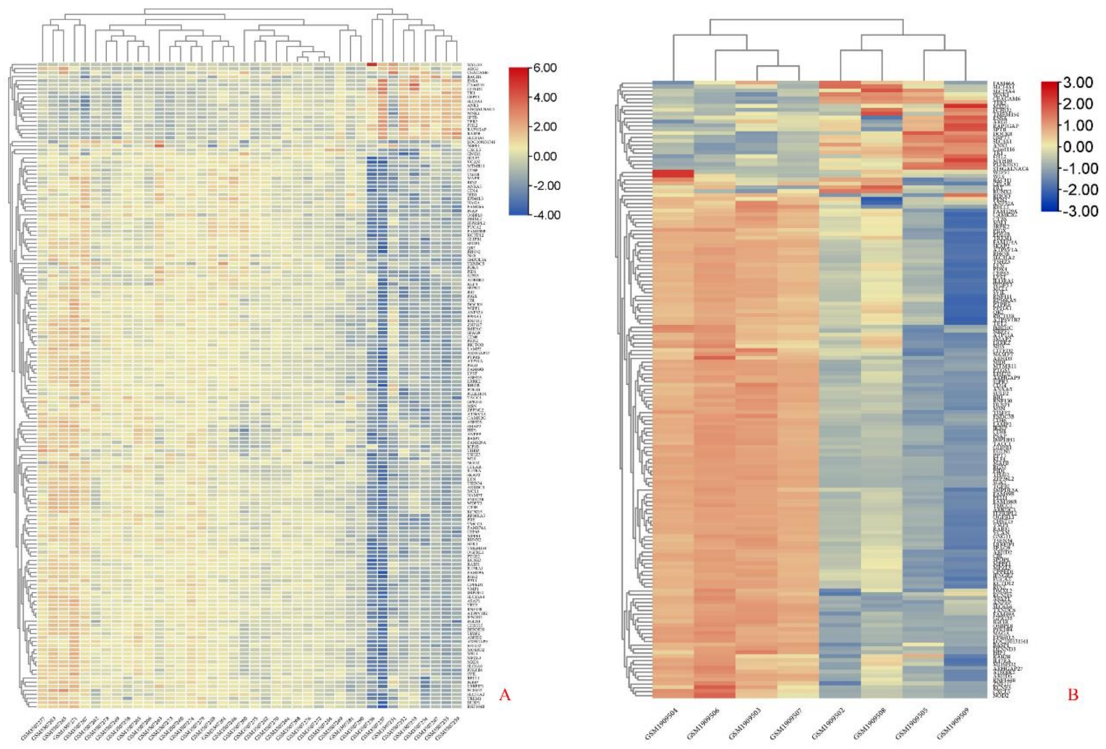

Supplementary Fig. S1 Heatmaps of the DEGs between ONFH patients and normal people. **A)** based on data from GSE123568; **B)** based on data from GSE74089.

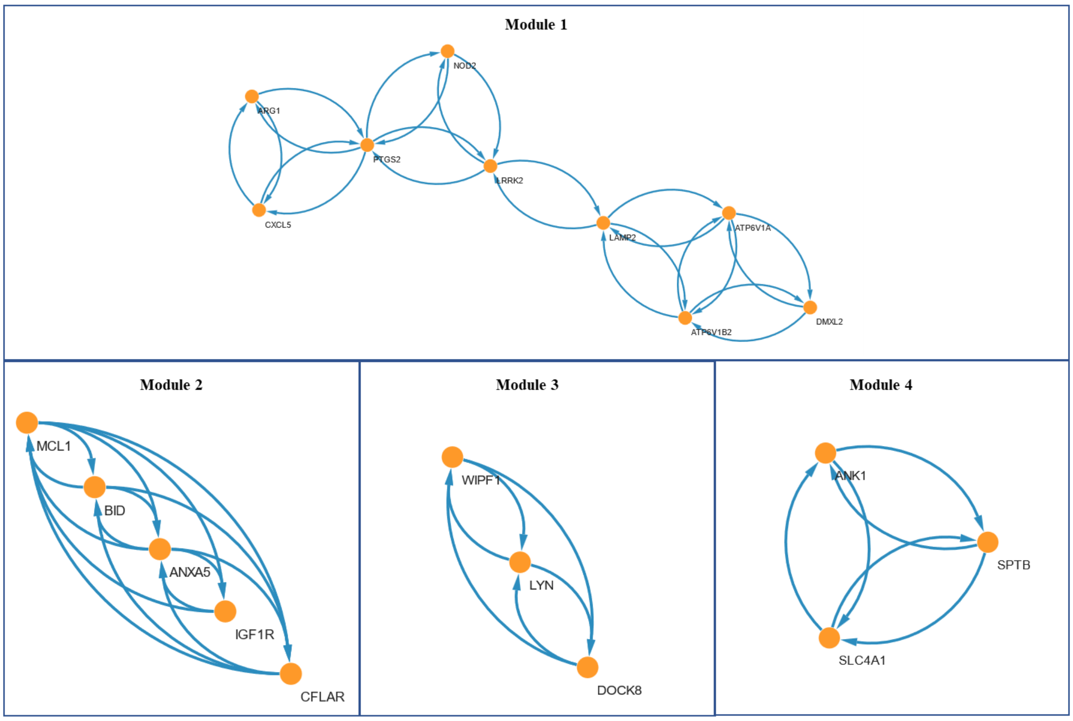

Supplementary Fig. S2 PPI network of the top 4 functional clusters of modules constructed in Cytoscape software. Module 1 consisted of 9 nodes and 24 edges, and genes in module 1 were enriched in the TNF signaling pathway, collecting duct acid secretion, synaptic vesicle cycle, and IL-17 signaling pathway. Module 2 consisted of 5 nodes and 16 edges, and the KEGG pathway analysis results indicated that genes in module 1 were enriched in the apoptosis. Module 3 consisted of 3 nodes and 6 edges, and genes in module 3 were enriched in the SH3 domain binding. Module 4 consisted of 3 nodes and 6 edges, and genes in module 4 were enriched in the spectrin-associated cytoskeleton, cortical cytoskeleton, and cytoplasmic side of plasma membrane.
